# In-vivo monitoring of anti-folate therapy in arthritic rats using [^18^F]fluoro-PEG-folate and positron emission tomography

**DOI:** 10.1186/s13075-017-1325-x

**Published:** 2017-05-31

**Authors:** Durga M. S. H. Chandrupatla, Gerrit Jansen, Ricardo Vos, Mariska Verlaan, Qingshou Chen, Philip S. Low, Albert D. Windhorst, Adriaan A. Lammertsma, Conny J. van der Laken, Carla F. M. Molthoff

**Affiliations:** 1 Amsterdam Rheumatology and Immunology Center, VU University Medical Center, De Boelelaan 1117, 1081 HV Amsterdam, The Netherlands; 2 Department of Radiology & Nuclear Medicine, VU University Medical Center, De Boelelaan 1117, 1081 HV Amsterdam, The Netherlands; 3 Department of Chemistry, Purdue University, 720 Clinic Drive, West Lafayette, IN 47907-2084 USA

**Keywords:** [^18^F]fluoro-PEG-folate, Folate receptor β, Methotrexate, Rheumatoid arthritis, Macrophages

## Abstract

**Background:**

Folate receptor β (FRβ) is involved in facilitating cellular uptake of folates and anti-folates (such as methotrexate (MTX)). In rheumatoid arthritis, FRβ is expressed on synovial macrophages and recently has been explored as a biomarker for imaging in arthritic rats using the folate-based positron emission tomography (PET) tracer [^18^F]fluoro-PEG-folate. The purpose of this study was to examine whether this folate tracer can also be used to monitor therapeutic efficacy of MTX in arthritic rats.

**Methods:**

Arthritic rats received either no treatment or MTX therapy (1 mg/kg, either 2× or 4×). Healthy rats did not receive any arthritic induction or therapy. [^18^F]fluoro-PEG-folate PET-CT scans (60 min) were performed before and after MTX therapy. Following PET, the ex-vivo tissue distribution of radioactivity was determined in excised knees and multiple tissues. Synovial macrophage infiltration in knee sections was quantified by immunohistochemistry using ED1 and ED2 antibodies.

**Results:**

PET scans clearly visualized increased uptake of [^18^F]fluoro-PEG*-*folate in arthritic knees compared with contralateral knees. Significantly lower standard uptake values (1.5-fold, *p* < 0.01) were observed in arthritic knees of both MTX-treated groups after therapy, approximating the levels seen in healthy rats. Consistently, e*x*-vivo tissue distribution demonstrated a 2–4-fold lower tracer uptake in the arthritic knee of 2× and 4× MTX-treated rats, respectively, compared with control rats. These results were corroborated with significantly reduced (2–4-fold, *p* < 0.01) ED1-positive and ED2-positive synovial macrophages in arthritic knees of the MTX-treated rats compared with those of the control rats.

**Conclusion:**

This study in arthritic rats underscores the potential and usefulness of [^18^F]fluoro-PEG*-*folate PET as a therapeutic monitoring tool of MTX therapy and potentially other anti-folate treatment of arthritis.

## Background

Methotrexate (MTX) is the anchor drug in rheumatoid arthritis (RA) therapy, either as a single agent or in combination with disease-modifying anti-rheumatic drugs and biological agents [1–4]. Membrane transport via carrier-mediated and receptor-mediated routes is the first regulatory step in the mechanism of action of MTX in immunological target cells [5–7]. Notably, in RA (synovial) macrophages, the folate receptor β (FRβ) has been recognized as a major transport route for MTX, next to the reduced folate carrier [8, 9]. FRβ expression is confined to cells of the myeloid lineage [10, 11] as opposed to the α-isoform of FR (FRα), which is selectively expressed in specific types of cancer (ovary, breast) [12–14]. Given the high binding affinity (low nanomolar Kd) of FR for folic acid, this receptor has been exploited for therapeutic targeting with folate-conjugated drugs [15, 16] as well as imaging of FRα-positive tumours and activated FRβ-positive macrophages in RA [17–19]. FRs harbour several interesting properties for targeting with folate-based positron emission tomography (PET) tracers; for example, easy accessibility as an extracellular GPI-anchored membrane protein, high binding affinities for folates, and specific expression on activated macrophages in inflammatory diseases, allowing receptor targeting for imaging with folate-based PET tracers [9, 10].

In humans, macrophages have been identified as a sensitive biomarker for therapy monitoring, regardless of the choice of treatment [20]. Moreover, RA remission has been positively correlated with lower numbers of synovial macrophages [21]. These findings, however, were obtained by invasive histological studies. Clearly, non-invasive imaging of macrophages may be an attractive alternative approach to detect and monitor synovial activity in body tissues [22]. Animal models of arthritis can serve as a pre-clinical step to explore macrophage expression through novel imaging modalities. Beyond successful application of single-photon imaging agents—for example, EC20, a ^99m^Tc-labelled folate [23–25]—to image arthritis, recently a folate-based PET tracer, [^18^F]fluoro-PEG-folate, has been synthesized [26]. Such a tracer could potentially employ the higher sensitivity of PET and its ability to quantify uptake, which is essential for intervention studies. The potential of [^18^F]fluoro-PEG-folate for imaging macrophages has been demonstrated in an arthritic animal model [26]. However, the potential of [^18^F]fluoro-PEG-folate to monitor the efficacy of therapeutic interventions, in particular using anti-folates, such as MTX, has not been explored.

In the present study, the potential of [^18^F]fluoro-PEG-folate as a macrophage-targeted PET agent for monitoring MTX therapy efficacy in arthritic rats was examined.

## Methods

### Animals

Wistar rats (male, 150–200 g; Charles River International Inc, Sulzfeld, Germany) were provided with standard food (16% protein rodent diet; Harlan Laboratories Inc., Madison, WI, USA) and water ad libitum. Rats were housed in groups of three to six in conventional cages and kept in a room with a 12-hour light/dark cycle, and constant room temperature (20 °C) and humidity level (50%). Animal experiments were performed in accordance with the European Community Council Directive 2010/63/EU for laboratory animal care and the Dutch Law on animal experimentation. The experimental protocol was validated and approved by the local committee on animal experimentation of the VU University Medical Center (DEC PET13-07).

### Arthritic induction and therapeutic interventions

**Fig. 1:**
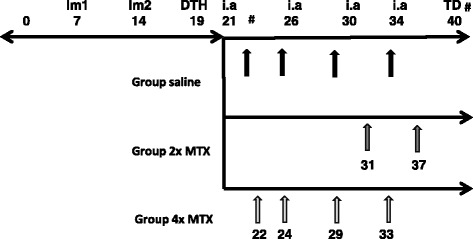
Timeline depicting the induction of arthritis in rats and thereupon methotrexate (*MTX*) interventions. On day 7 and day 14 the first and second immunization (*Im1* and *Im2*) was administered, on day 19 the delayed type hypersensitivity (*DTH*) test and thereupon four intra-articular (*i.a*.) injections were administered. Upon arthritic induction the rats were administered saline (*black arrows*) or 2× MTX (*dark grey arrows*) or 4× MTX (*light grey arrows*). PET-CT (*#*) was performed before (day 22) and after (day 40) MTX therapy. At the end of the study (day 40), ex-vivo tissue distribution (*TD*) was performed. Healthy rats did not receive any arthritic induction or MTX treatment and were sacrificed on day 40

Wistar rats received 4× intra-articular (i.a.) mBSA injections, 4 or 5 days apart, in the arthritic knee as described previously [27]. This model had resemblance to human arthritis because after immunization and an i.a. mBSA injection in one knee, arthritis develops within a week as manifested by an increased knee thickness and synovial macrophage infiltration in the arthritic vs contralateral knee. This adds another major advantage to this model because the contralateral knee serves as an internal control over the arthritic knee. Additionally, by applying successive intra-articular mBSA injections after the first i.a. mBSA injection, a prolonged chronic phase of arthritis is maintained allowing assessments of therapeutic interventions. MTX (VU University Medical Center’s pharmacy, the Netherlands) was administered (1 mg/kg) intraperitoneally (i.p.) once at days 22, 24, 29 and 33 (after the first i.a. injection) for the 4 × -MTX group (*n* = 4 rats) or once 3 days before (day 31) and once 3 days after (day 37) the fourth i.a. injections (2 × -MTX group, *n* = 4). Control rats (untreated control group, *n* = 4 rats) received phosphate-buffered saline (PBS) i.p. once at days 22, 24, 29 and 33 (after the first i.a injection). Healthy rats did not undergo arthritis induction or receive any treatment. At the end of therapy (~day 40), all rats were sacrificed and tissues were excised for further processing. Figure 1 summarizes the schedule of arthritis induction, therapeutic interventions and various analyses.

### [^18^F]fluoro-PEG-folate synthesis and PET-CT

[^18^F]fluoro-PEG-folate was synthesized as described previously [26], with a radiochemical purity > 97% and mean specific activity of 31.4 ± 5.5 GBq/μmol. Rats were anaesthetized using inhalation anaesthetics (isoflurane 2–2.5% and oxygen 0.45 volume %). The tail vein was cannulated with a poly-urethane 3-French cannula. During PET-CT (Mediso nanoPET-CT, Budapest, Hungary) rats were placed in an integrated heating bed while monitoring respiratory function. A computed tomography (CT) scan was performed for 5 min, followed by tracer administration (10.5 ± 1.1 MBq) at the start of a dynamic PET scan of 60 min. PET data were normalized, and corrected for scatter, randomization, attenuation, decay and dead time. List-mode PET data were re-binned in 19 successive frames (4 × 5, 4 × 10, 2 × 30, 3 × 60, 2 × 300, 3 × 600 and 1 × 900 s), which were reconstructed using an iterative 3D Poisson ordered-subsets expectation-maximization algorithm with four iterations and six subsets. Resulting images had a matrix size of 170 × 170 × 157 voxels, each with a dimension of 0.6 × 0.6 × 0.6 mm^3^.

### PET data analysis

Images were analysed using AMIDE software (A Medical Image Data Examiner, version 0.9.2) [28]. CT and PET images were superimposed for drawing regions of interest (ROI). Fixed-size ellipsoidal-shaped ROIs (dimensions: 7 × 4 × 7 mm^3^) were drawn manually over the area of both arthritic and contralateral knees in the last frame. Next, ROIs were projected onto the dynamic image sequence and time–activity curves (TACs) were generated. TACs were expressed as standardized uptake values (SUV); that is, mean ROI radioactivity concentration normalized to injected dose and body weight.

### Ex-vivo tissue distribution studies

All rats were sacrificed (60 min after tracer administration) and knees, blood and various internal organs were excised, rinsed, dipped dry, weighed and the amount of radioactivity determined using an LKB 1282 Compugamma CS gamma counter (LKB, Wallac, Turku, Finland). Results were expressed as percentage of the injected dose per gram of tissue (%ID/g) [27].

### Histopathology and immunohistochemistry

Both knees were dissected in toto and fixed for 7 days at 4 °C in 10% freshly made paraformaldehyde in PBS with 2% sucrose (pH 7.3) prior to decalcification in osteosoft (101728; Merck, Darmstadt, Germany) for ~2.5 weeks at room temperature. Thereafter, knees were embedded in paraffin. Sections of 5 μm were cut through the centre of the joint in a longitudinal direction and stained with haematoxylin and eosin to assess the degree of inflammation in synovial tissue. Staining for macrophages was performed as described previously [27]. Briefly, after antigen retrieval, sections were incubated with the specific mouse anti-rat monoclonal antibodies ED1, homologous to human CD68, and ED2, homologous to human CD163, or isotype control antibody for 1 hour at RT. All antibodies were obtained from Hycult (Plymouth Meeting, PA, USA). The detection EnVision™ kit (K4063 dual-link-HRP rabbit/mouse; DAKO, Glostrup, UK) was used according to the instructions of the manufacturer with 3,3′-diaminobenzidine tetrahydrochloride (DAB; DAKO) containing 0.01% H_2_O_2_. Subsequently, sections were counterstained with haematoxylin, dehydrated and mounted. Images were captured using a Leica 4000B microscope and Leica digital camera DC500 (Microsystems B.V. Rijswijk, the Netherlands).

All stained slides were blinded and counted by two independent observers, guided by an experienced pathologist, for ED1-positive and ED2-positive synovial macrophages. For this purpose, knee sections were divided into four quadrants (Q1–Q4), each representing the joint capsule with synovial tissue lining on either side of the proximal and distal side of the bone. Under the microscope (Leica, Amsterdam, the Netherlands) at 400× magnification, in each quadrant two to three areas were evaluated for macrophages in the lining and sub-lining (1–10 layers) of the synovium. The average number of macrophages per area from all four quadrants were combined and presented as total number of ED1 or ED2 macrophages (± standard deviation (SD)).

### Statistical analysis

Statistical analysis was performed using SPSS (version 15) for Windows (SPSS Inc., Chicago, IL, USA). The Wilcoxon signed-rank (exact) test was used to determine differences in paired observations, such as uptake of [^18^F]fluoro-PEG-folate in arthritic versus contralateral knees. Mann–Whitney (exact) tests were performed to analyse differences in [^18^F]fluoro-PEG-folate uptake in groups (i.e. arthritic versus normal and control knees). *p* < 0.05 was considered statistically significant. All results are represented as mean ± SD.

## Results

### Arthritis induction and therapeutic interventions

Arthritis induction in rats (see Fig. 1 for timeline) was associated with macroscopic thickening of the arthritic knee compared with the contralateral knee (data not shown). Therapeutic interventions with MTX at the time points depicted in Fig. 1 were not associated with any adverse effects or visible effects on knees and no significant changes in body weight were observed.

### [^18^F]fluoro-PEG*-*folate PET studies in untreated and MTX-treated arthritic rats

**Fig. 2:**
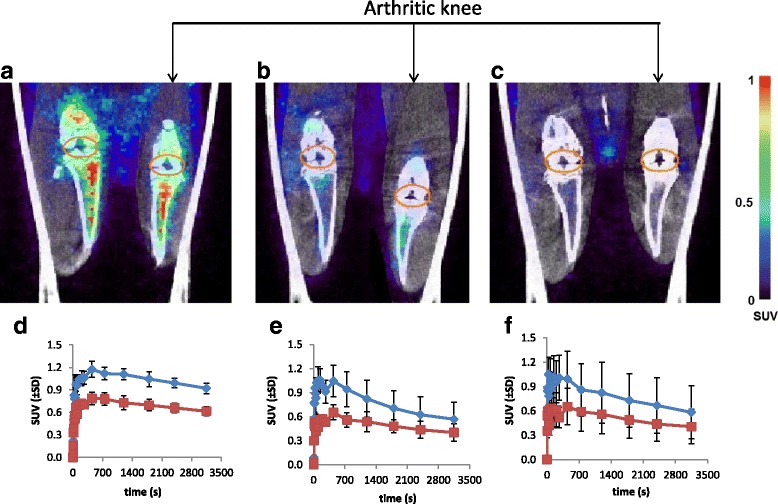
Representative coronal PET-CT scans of [^18^F]fluoro*-*PEG*-*folate in control and arthritic rats. **a** Control (before therapy, day 22); **b** 2× MTX and **c** 4× MTX (after therapy, day 40). *Orange ellipsoid*: ROI drawn around the synovium of the knee joint. Arthritic (*right*) and contralateral knees (*left*) depicted on each image. Standardized uptake value (*SUV*) scale bar from minimum 0 to maximum 1, represent the uptake of the tracer. TACs of [^18^F]fluoro*-*PEG*-*folate uptake are expressed as SUV (±SD) in arthritic and contralateral knees of the control group (**d**), 2 × -MTX group (**e**) and 4 × -MTX group (**f**)

At baseline, [^18^F]fluoro-PEG*-*folate PET scans (Fig. 2a–c) clearly visualized high uptake in the arthritic knee of control rats, which decreased in both the 2 × -MTX and 4 × -MTX groups. Before treatment, [^18^F]fluoro-PEG*-*folate SUV in the arthritic knee (1.01 ± 0.07) was significantly (*p* < 0.01) higher than in the contralateral knee (0.67 ± 0.04) (Fig. 2a, d). After MTX treatment, both the 2 × -MTX (0.67 ± 0.11) (Fig. 2b) and 4 × -MTX (0.70 ± 0.10) groups (Fig. 2c) showed a significantly (*p* < 0.01) 1.5-fold lower uptake of [^18^F]fluoro-PEG*-*folate compared with untreated arthritic rats. In fact, SUV values in the arthritic knee of both 2 × -MTX and 4 × -MTX rats (Fig. 2e, f) were comparable with the level of uptake in knees of healthy rats (0.67 ± 0.07) (data not shown).

### Ex-vivo tissue distribution studies

**Fig. 3:**
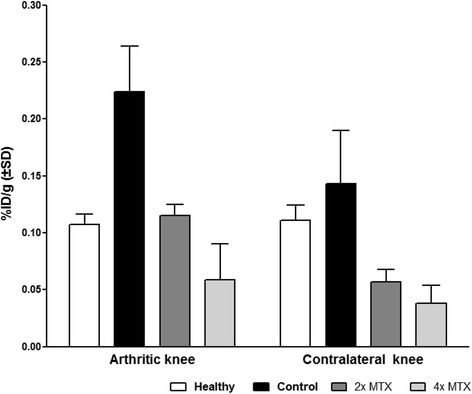
Ex-vivo tissue distribution of [^18^F]fluoro*-*PEG*-*folate in arthritic and contralateral knees of healthy (*white bars*), control rats (*black bars*) and 2 × -MTX (*dark grey*) and 4 × -MTX (*light grey*) rats at 60 min post tracer injection. Results expressed as mean percentage injected dose per gram (*%ID/g*). *Error bars* indicate SD. *MTX* methotrexate

Before treatment, control rats showed a significantly 1.5-fold (*p* < 0.05) higher uptake (expressed as %ID/g) of [^18^F]fluoro-PEG*-*folate in arthritic knees (0.22 ± 0.04) compared with contralateral knees (0.14 ± 0.04). After MTX treatment, [^18^F]fluoro*-*PEG*-*folate uptake in arthritic knees was significantly 2-fold (*p* < 0.05) lower in the 2 × -MTX group (0.11 ± 0.01) and 4-fold (p < 0.01) lower in the 4 × -MTX group (0.06 ± 0.03) compared with untreated arthritic rats (Fig. 3).

Uptake of [^18^F]fluoro-PEG*-*folate in plasma did not differ between control vs 2 × -MTX and 4 × -MTX rats (0.010 ± 0.006 vs 0.009 ± 0.001 and 0.009 ± 0.001, respectively) (Table 1). MTX treatment, however, did reduce [^18^F]fluoro-PEG-folate uptake in high macrophage resident organs, as illustrated in Table 1 for the control group vs both 2 × -MTX and 4 × -MTX groups in the liver, heart, spleen, lung and bone. In the kidney and intestine, MTX treatment had no major impact on tracer uptake in control, 2 × -MTX and 4 × -MTX rats (Table 1).

**Table 1:** Ex-vivo tissue distribution of [^18^F]fluoro*-*PEG*-*folate in various tissues of healthy control, 2 × -MTX and 4 × -MTX rats at 60 min post tracer injection

Tissue	Healthy rats	Control rats	2 × -MTX group	4 × -MTX group
Bone	0.103 ± 0.040	0.134 ± 0.022	0.064 ± 0.011	0.032 ± 0.016
Plasma	0.013 ± 0.007	0.010 ± 0.006	0.009 ± 0.002	0.010 ± 0.001
Blood	0.007 ± 0.009	0.007 ± 0.001	0.005 ± 0.002	0.006 ± 0.001
Lung	0.051 ± 0.019	0.074 ± 0.024	0.025 ± 0.016	0.024 ± 0.005
Heart	0.045 ± 0.015	0.080 ± 0.028	0.034 ± 0.004	0.022 ± 0.006
Liver	0.081 ± 0.049	0.106 ± 0.041	0.053 ± 0.006	0.042 ± 0.01
Spleen	0.393 ± 0.131	0.551 ± 0.218	0.132 ± 0.027	0.062 ± 0.034
Kidney	3.106 ± 0.317	2.077 ± 0.895	3.386 ± 0.186	3.098 ± 0.612
Skin	0.116 ± 0.017	0.138 ± 0.025	0.090 ± 0.012	0.063 ± 0.027

Results expressed as mean percentage injected dose per gram (%ID/g) ± standard deviation

*MTX* methotrexate

### Immunohistochemistry of synovial macrophages

**Fig. 4:**
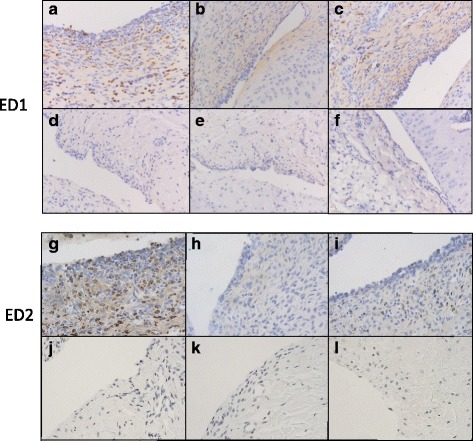
Representative images of ED1^+^ and ED2^+^ synovial macrophages in knee sections of control and MTX-treated rats. **a**, **b**, **c** Images represent ED1^+^ synovial macrophages in the arthritic knee of control, 2 × -MTX and 4 × -MTX rats, respectively. **d**, **e**, **f** ED1^+^ synovial macrophages in the contralateral knee of control, 2 × -MTX and 4 × -MTX rats, respectively. **g**, **h**, **i** images of ED2^+^ synovial macrophages in the arthritic knee of control and 2 × -MTX and 4 × -MTX rats, respectively. **j**, **k**, **l** ED2^+^ synovial macrophages in the contralateral knee of control, 2 × -MTX and 4 × -MTX rats, respectively. All images captured at 200× magnification

**Fig. 5:**
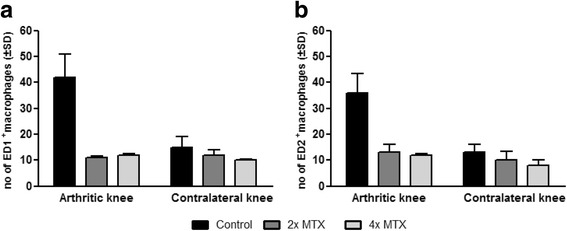
Quantification of ED1^+^ and ED2^+^ synovial macrophages in knee sections of control and treated rats. **a** ED1^+^ synovial macrophages, 6 days after the third boost, of control rats (*black bars*) and 2 × -MTX (*dark grey*) and 4 × -MTX (*light grey*) rats. **b** ED2^+^ synovial macrophages, 6 days after the third boost, of control rats (*black bars*) and 2 × -MTX (*dark grey*) and 4 × -MTX (*light grey*) rats. Values represent mean number of macrophages counted in predefined areas of the synovium. *Error bars* indicate SD. *MTX* methotrexate, *SD* standard deviation

To examine whether the lower [^18^F]fluoro-PEG-folate uptake in arthritic knees after MTX treatment was due to reduced infiltration of synovial macrophages, ED1-positive and ED2-positive macrophages in synovial tissue were quantified. Microscopically, synovial tissue of arthritic rats showed cellular influx of ED1^+^ and ED2^+^ macrophages (Fig. 4a–l), the latter of which were significantly (*p* < 0.01) more abundant: 3-fold (42 ± 9 vs 15 ± 4) and 2-fold (36 ± 8 vs 13 ± 3), respectively, in the arthritic and contralateral knees (Figs. 4a vs d, g vs j and 5). Both the 2 × -MTX and 4 × -MTX treatment groups of arthritic rats showed a marked and significant reduction of ED1^+^ and ED2^+^ synovial macrophages in the arthritic knees compared with the untreated counterparts (Fig. 4). Quantification showed a 4-fold and 3-fold (*p* < 0.01) reduction in ED1^+^ synovial macrophages after 2 × -MTX and 4 × -MTX treatments (Fig. 5a), respectively. For ED2^+^ the reduction was 3-fold (*p* < 0.01) for both groups (Fig. 5b). Notably, MTX treatment reduced ED1^+^ and ED2^+^ synovial macrophages in arthritic knees to levels observed in contralateral knees (Fig. 5).

## Discussion

The present study, using [^18^F]fluoro-PEG*-*folate, investigated the feasibility of non-invasively monitoring efficacy of anti-folate therapeutic interventions in RA. Lower accumulation of [^18^F]fluoro-PEG*-*folate in arthritic knees corroborated with decreased numbers of active macrophages in MTX-treated rats compared with the untreated rats. This was illustrated for MTX, because this is the golden standard in clinically active RA treatment [1, 3, 4].

Folate receptor expression on activated macrophages has been exploited for imaging and therapeutic monitoring of arthritis with various folate PET tracers including 4-[^18^F]fluorophenylfolate and [^68^Ga]-DOTA-folate [29]. These PET tracers showed a significantly improved specificity over a general inflammation tracer [^18^F]-FDG, which relates to increased glucose metabolism in, for example, activated macrophages. In the present study, we made use of a pegylated folate tracer, [^18^F]fluoro-PEG*-*folate, which harbours improved plasma pharmacokinetic properties over other folate tracers. In a side-by-side comparison in a rat model for RA [27], [^18^F]fluoro-PEG*-*folate demonstrated a 1.5× improved target to background ratio compared with the mitochondrial translocator protein targeted macrophage tracer (*R*)-[^11^C]PK11195 [26]. Moreover, [^18^F]fluoro-PEG*-*folate also displayed promising PET imaging potential [26], which was taken a step further in the present study for monitoring therapeutic interventions, such as MTX therapy.

[^18^F]fluoro-PEG*-*folate PET combined with a CT has advantage over the previous reported [^18^F]fluoro-PEG*-*folate PET study [26], because the region of interest (ROI) around the synovium can be depicted more precisely. [^18^F]fluoro-PEG*-*folate showed a marked reduction in tracer uptake in arthritic knees of the rats following two different MTX treatment regimens. It is unlikely that reduced tracer uptake in the MTX-treated rats is due to direct competition of the radiolabelled tracer with MTX for FRβ for various reasons: PET scans were acquired in the last week after the last MTX dose and, based on MTX pharmacokinetics [30] at that time, residual plasma levels will be <10 nM; the binding affinity of FRβ for [^18^F]fluoro-PEG*-*folate outweighs the binding affinity for MTX by at least 100-fold; and also the binding affinity of the natural circulating plasma folate (i.e. 5-methyltetrahydrofolate) is 3-fold higher than the tracer [9, 26], and thus competitive effects are not anticipated. In addition, immunohistochemical analysis of the arthritic joints showed a significant reduction of macrophages in synovial tissue which was in line with reduced joint uptake of the folate tracer. Consistent with our PET results, Kelderhouse et al. [31] also demonstrated a markedly lower accumulation of the SPECT folate targeted imaging agent [^99m^Tc]-EC20 in a collagen-induced arthritis (CIA) model upon administration of anti-rheumatic drugs. In the same CIA model, OTL0038, a novel folate-conjugated near-infrared dye, also showed low accumulation following anti-rheumatic therapies [32]. Together, whereas SPECT and optical imaging each has proven value with folate-based imaging agents, PET folate harbours advantages over SPECT (low-resolution and low-sensitivity images) [26] and optical imaging (no deep tissue imaging) [32]. Although costs of PET are relatively high at this moment, it is anticipated that with the widespread application of PET technology worldwide, costs will come down in the near future as also happened in the past decennia for the other imaging techniques such as CT and MRI.

Previously, apart from prominent arthritis induction in the arthritic knee, signs of systemic inflammation were also observed [27], reflected by tracer uptake in macrophage-rich organs, especially the liver and spleen. Ex-vivo tissue distribution data indicated that MTX therapy also had systemic effects by reducing [^18^F]fluoro-PEG*-*folate uptake in these organs as well as in the contralateral knee. Independent of MTX therapy, the increased accumulation of [^18^F]fluoro-PEG*-*folate in kidneys and intestinal tissue could be attributed to tracer clearance and/or high expression of FRα on kidney proximal tubule cells [15, 17] and intestinal tissue [33] to which receptor the folate tracer also binds.

Immunohistochemical analyses indicated that markedly reduced numbers of macrophages in the synovium of MTX-treated arthritic rats accounted for reduced [^18^F]fluoro-PEG*-*folate tracer uptake. Interestingly, reduction in macrophages upon MTX treatment involved both ED1-positive and ED2-positive macrophages—although CD68 (and possibly its rat homologue ED1 used in the present study) is not an absolute marker for macrophages because its expression has also been demonstrated on fibroblasts. To extend on this point, an experienced pathologist examined the (arthritic) knee sections for morphological assessment of cellularity and immunohistochemical staining of ED1^+^ cells. ED1^+^ synovial fibroblasts were identified but morphologically discerned from macrophages and were not taken into account for macrophage scoring assessments. Moreover, from the perspective of macrophage polarization, we also examined numbers of ED2^+^ macrophages (the rat homologue of human CD163, so-called ‘M2’, ‘anti-inflammatory macrophages’) in synovial tissue before and after therapy. Notably, studies by Puig-Kroger et al. [34] showed that FRβ is differentially expressed on M2 macrophages upon ex-vivo skewing of monocytes with macrophage-colony stimulating factor. However, studies by Tsuneyoshi et al. [35] in human RA synovial tissue demonstrated mixed patterns of FRβ expression on ‘M1’ (‘pro-inflammatory’) and M2 macrophages. In this context it is also important to note that M2 macrophages in an RA environment with complex IgG autoantibodies and/or ACPA antibodies are triggered to produce pro-inflammatory cytokines [36, 37]. Thus, MTX targeting with respect to polarization of macrophages and the role of FRβ therein warrant further investigations. Given the fact that beyond MTX several other second-generation anti-folates have been developed which have demonstrated potential pre-clinical anti-arthritic activity [7, 9, 38–40], [^18^F]fluoro-PEG*-*folate PET imaging may be useful for monitoring their efficacy.

## Conclusion

The present study demonstrates the feasibility of in-vivo monitoring of MTX therapy in arthritic rats using [^18^F]fluoro-PEG*-*folate PET, paving the way for its future use in human clinical RA.

## Abbreviations

FDG: FluoroDeoxyGlucose
FRβ: Folate receptor beta
i.a.: Intra-articular
ID/g: Injected dose/gram
M-CSF: Macrophage-colony stimulating factor
MTX: Methotrexate
PEG: Polyethylene glycol
PET-CT: Positron emission tomography–computer tomography
RA: Rheumatoid arthritis
ROI: Region of interest
SD: Standard deviation
SUV: Standardized uptake value
